# Supplementary material for: Psychological profiles of South African smallholder farmers
Source: PLoS One. 2023 Feb 21;18(2):e0265634. doi: 10.1371/journal.pone.0265634 (PMC9943007; doi:10.1371/journal.pone.0265634)
Supplement: Attachment — Submitted filename: 4. Response to Reviewers FINAL.docx [file pone.0265634.s001.docx]

Dear Editor

Please find below each reviewer’s comment followed by an explanation of the revisions made in response to the comment.

**Review Comments to the Author**

**Reviewer #1:**

1. The topic addressed in the paper is of high importance from the point of view of the South African economy - as indicated in the paper; however, the research question, and the findings might be generalisable or referred to by other countries as well.

**Response:** Thank you for your suggestion. However, given the characteristics of the sample of smallholder farmers in South Africa, we are cautious in making any such claim about the generalisability of our findings. We have now noted that our findings could be replicated in different contexts and other countries in the African continent at p. 29-30.

2. The paper clearly describes the current situation, provides necessary information regarding the research sample and the results and introduces the reader to a detailed analysis of the data.

**Response:** Thank you.

3. The conclusions are in line with the findings presented.

**Response:** Thank you.

4. The paper addresses plenty of international literature, some of which are rather old. Nonetheless, there are some (5) papers from the past five years that underline the topicality of the paper. I would recommend the authors to search for some fresh literature to replace the old ones, especially regarding the applied methodology.

**Response:** We have now included further relevant research literature pertaining to key psychological predictors of farm business performance at p. 5.

**Reviewer #2:**

1. The paper focuses a really challenging and actual topic. Identifying the barriers of engaging agricultural production is essential information for both the academic community and decision makers. The psychological approach is among the most important key factors indeed.

**Response:** Thank you for the positive feedback.

2. The title of the paper is sound and focusing appropriately, covering and reflecting the content. The abstract is well written and well understandable. Keywords are well selected.

**Response:** Thank you.

3. The introduction is acceptable, highlighting the importance of the topic and the context in a proper way.

**Response:** Thank you.

4. I am not satisfied with the literature review. Some essential part of the sources are overviewed but in fact, only those which were used to determine the methodology an setting the context. However, the "real" literature overview, where the works of other authors who are (were) dealing with the same topic is missing. I recommend to set a critical, analytical and comparative literature review after the introduction part.

**Response:** As suggested, we have now revised the literature review section to provide an overview of literature examining an association between key psychological variables and farm business performance (see p. 5).

5. The methodology description is well written, the results are supported by the methods.

**Response:** Thank you.

The conclusion part is unacceptable short, the authors should extend it, based on the results.

**Response:** As suggested, we have now included additional information at p. 29-30.

**Journal Requirements:**

When submitting your revision, we need you to address these additional requirements.

1. Please ensure that your manuscript meets PLOS ONE's style requirements, including those for file naming.

**Response:**

2. We note that you have referenced (Bhullar, N. (2021)) which has currently not yet been accepted for publication. Please remove this from your References and amend this to state in the body of your manuscript: (Bhullar, N. (2021). [Unpublished]”) as detailed online in our guide for authors [http://journals.plos.org/plosone/s/submission-guidelines#loc-reference-style](https://aus01.safelinks.protection.outlook.com/?url=http%3A%2F%2Fjournals.plos.org%2Fplosone%2Fs%2Fsubmission-guidelines%23loc-reference-style&data=04%7C01%7Cnbhulla2%40myune.mail.onmicrosoft.com%7C0d5bd20bab5242274e5508d9cf45d7af%7C3e104c4f8ef24d1483d8bd7d3b46b8db%7C0%7C0%7C637768722095534255%7CUnknown%7CTWFpbGZsb3d8eyJWIjoiMC4wLjAwMDAiLCJQIjoiV2luMzIiLCJBTiI6Ik1haWwiLCJXVCI6Mn0%3D%7C3000&sdata=qidp1dgVdqzOp1O0A326EBNW1z2xFeSmuzPYIrfE%2Bw0%3D&reserved=0).

**Response:** Thank you. We have now revised the manuscript text.

3. Thank you for stating the following in the Acknowledgments Section of your manuscript:

“Research undertaken with beef farmers in this study was funded by the Australian Centre for International Agricultural Research [grant number LS/2016/276]. Data derived from poultry farmers in this study was sourced through South Africa’s Poultry Improvement and Recording Scheme, administered by the Agricultural Research Council and funded by the South African Department of Agriculture, Land and Rural Development.”

We note that you have provided additional information within the Acknowledgements Section that is not currently declared in your Funding Statement. Please note that funding information should not appear in the Acknowledgments section or other areas of your manuscript. We will only publish funding information present in the Funding Statement section of the online submission form.

Please remove any funding-related text from the manuscript and let us know how you would like to update your Funding Statement. Currently, your Funding Statement reads as follows:

“Initials of the authors who received each award:

HMB for the ACIAR project and NBN for the DALRRD funds

Grant numbers awarded to each author:

ACIAR LPS/2016/276 and no grant number for the DALRRD funds

The full name of each funder:

Australian Centre for International Agricultural Research; South African Department of Agriculture, Land Reform and Rural Development

URL of each funder website:

<https://www.aciar.gov.au/>   <https://www.dalrrd.gov.za/>  <https://www.arc.agric.za/arc-api/Pages/Animal-Recording-and-Improvement.aspx> (ARC is the administrator of DALRRD funds for the poultry recording scheme)”

Please include your amended statements within your cover letter; we will change the online submission form on your behalf.

**Response:** Thank you. We have removed funding information provided in the manuscript.

4. Please review your reference list to ensure that it is complete and correct. If you have cited papers that have been retracted, please include the rationale for doing so in the manuscript text, or remove these references and replace them with relevant current references. Any changes to the reference list should be mentioned in the rebuttal letter that accompanies your revised manuscript. If you need to cite a retracted article, indicate the article’s retracted status in the References list and also include a citation and full reference for the retraction notice.

**Response:** Thank you, we have checked the Reference list for accuracy.
